# Ki-67 as a Prognostic Marker in Squamous Cell Carcinomas of the Vulva: A Systematic Review

**DOI:** 10.3390/jcm14062045

**Published:** 2025-03-17

**Authors:** Gilbert Georg Klamminger, Elke Eltze, Annick Bitterlich, Yaman Degirmenci, Annette Hasenburg, Mathias Wagner, Meletios P. Nigdelis

**Affiliations:** 1 Department of Obstetrics and Gynecology, University Medical Center of the Johannes Gutenberg University Mainz, 55131 Mainz, Germany; 2 Department of General and Special Pathology, Saarland University (USAAR), Saarland University Medical Center (UKS), 66421 Homburg, Germany; 3 Institute of Pathology, Saarbrücken-Rastpfuhl, 66113 Saarbrücken, Germany; 4 Department of Gynecology and Obstetrics, Saarland University Medical Center (UKS), 66421 Homburg, Germany

**Keywords:** ki-67, MIB-1, vulvar cancer, lymph node metastasis, survival

## Abstract

**Background/Objectives**: To evaluate the prognostic impact of immunohistochemical ki-67 staining analysis regarding lymph node involvement and survival data (overall/progression-free survival) in squamous cell carcinoma of the vulva. **Methods**: A systematic literature search of English and German articles was conducted (PubMed, Embase, Scopus, Web of Science) from 1980 to December 2023, including the search terms “vulvar Neoplasms”, “vulvar cancer”, “vulvar carcinoma”, “vulvar tumor”, ”vulvar tumour”, “vulvar malignancy”, “vulvar malignant”, “ki-67”, “MIB-1”, “MIB1”, “proliferative index”, “proliferative activity”, “mitotic index”, and “mitotic count”. Study quality was assessed using a two-step “mixed-criteria” approach; to synthesize study results, a narrative summary is provided. **Results**: In total, 13 studies were included in this systematic literature review. In general, two distinct methods of staining interpretation could be retrieved: A “pattern-based” method, as well as a cell count-based method. Ten of the included studies examined the relationship between ki-67 and lymph node involvement, nine studies included survival data as a parameter of interest; and only five studies defined both groin lymph node metastasis and survival data as outcome variables. While nine out of ten studies found no statistically significant association between ki-67 staining and lymph node metastasis, five out of nine studies determined an association between ki-67 status and overall survival, especially when employing a “pattern-based” method of staining interpretation. **Conclusions**: The prognostic value of ki-67 staining in terms of survival data has been reported ambivalently and should be subject to future studies. Furthermore, we did not find convincing evidence of an association between ki-67 and lymph node involvement.

## 1. Introduction

Since the ki-67 antibody was first described in 1983, its ability to estimate the proportion of cell growth has already proven to be particularly valuable in histopathological tumor diagnostics [1]. Labeling proliferative cells targeting the ki-67 (MKI67) nuclear protein, which is encoded by the MKI67 gene and exclusively expressed in cells that are in a cell cycle state outside of G0, it is feasible not only to determine the character of a neoplasia (benign/malignant), but also to determine the degree of differentiation (grading) in various tumor entities [2,3,4,5,6]. For example, the histopathological evaluation of the ki-67 proliferation index is integrated within the grading system of gastroenteropancreatic neuroendocrine neoplasms [7]. Particularly noteworthy is also the fact that ki-67 staining is related to overall survival in breast cancer patients and that several clinical applications are integrated within the standard routine: On the one hand, ki-67 is used to define the intrinsic subtype (luminal A / luminal B tumors) of breast cancer; on the other hand, it can be used (together with other parameters) for risk assessment and identification of patients who benefit from chemotherapeutical treatment in addition to endocrine therapy. More recently, its ability as a dynamic biomarker (following repeated measurements on both the biopsy material and the surgical specimen during short-term neoadjuvant endocrine therapy) has been described [8,9]. To date, joined efforts tackling interlaboratory inconsistencies aiming for standardized interpretation, as well as scoring of immunohistochemical data, culminated in the *International ki-67 in Breast Cancer Working Group* [10].

In addition to established risk factors (e.g., lymph node metastases) within HPV-associated/independent squamous cell carcinoma of the vulva, clinicopathological parameters of uncertain prognostic relevance, such as ulceration and perineural sheath infiltration, remain [11,12,13]. To date, neither the mode of immunohistochemical ki-67 detection (either analysis of different expression patterns or numeric quantity of positive cells) nor the prognostic associations with overall/progression-free survival or lymph node metastasis in vulvar cancer has been translated into clinical practice.

For this study, medical libraries were systematically screened for literature regarding ki-67 staining in vulvar cancers, and relevant study results were depicted to examine and summarize existing knowledge about the prognostic relevance of ki-67 immunohistochemistry with regard to lymph node metastasis and survival data in squamous cell carcinoma of the vulva.

## 2. Materials and Methods

This systematic review was conducted in line with the PRISMA guidelines (Preferred Reporting Items for Systematic Reviews and Meta-Analyses) using an a priori established protocol outlining methodological data screening and data collection [14]; institutional review board approval was waived, since only published studies were reviewed. For an additional overview, PICO questions to be answered by this systematic review are listed and summarized within the Supplementary Materials [15,16].

### 2.1. Systematic Search Strategy

The (bibliographic) databases MEDLINE, Scopus, Embase, and Web of Science were searched to identify reports of interest published between 1980 and 2023 (time of last consultation: December 2023), employing the search terms: “vulvar Neoplasms”, “vulvar cancer”, “vulvar carcinoma”, “vulvar tumor”, ”vulvar tumour”, “vulvar malignancy”, “vulvar malignant”, “ki-67”, “MIB-1”, “MIB1”, “proliferative index”, “proliferative activity”, “mitotic index”, and “mitotic count”, using the Boolean AND/OR operators. See Supplementary Table S1 for the individual search strategies and search terms listed for each database.

### 2.2. Inclusion and Exclusion Criteria

Studies applying the ki-67 antibody on HPV-associated/independent squamous cell carcinoma of the vulva were included (inclusion criteria); all studies not meeting the inclusion criteria, or which applied the antibody of interest on other tumor entities of the vulva, precursor lesions, inflammatory/autoimmune conditions such as lichen sclerosus, as well as articles in languages other than English or German and conference posters without publicity available data were excluded (exclusion criteria).

### 2.3. Outcomes

As outcome criteria, a reported association of ki-67 immunohistochemistry and the occurrence of lymph node metastasis and/or its association with overall/progression-free survival were defined.

### 2.4. Study Selection and Data Extraction

Duplicates were removed and title/abstract screening was performed to identify eligible reports via Rayyan© (Rayyan Systems, Inc, Cambridge, MA, USA) [17]. Consecutively, full texts were screened and relevant data (method of ki-67 quantification, outcome parameter of interest, method and result of underlying statistical analysis) was extracted and collected within an internal data sheet (Microsoft Office Word, version 16) by two authors (GGK, MPN).

### 2.5. Assessment of Study Quality and Risk of Bias

Initial assessment of study quality was employed using a “mixed-criteria” two-step screening approach (study participation, prognostic factor measurement, outcome measurement, analysis) in which eligible studies were screened for relevant quality items; see Supplementary Table S2 [18]. In order to systematically assess the risk of bias in each study, the Methodological Index for Non-Randomized Studies (MINORS) protocol was employed using 7 items: (1) definition of a clear aim, (2) inclusion of patients, (3) protocolized data collection, (4) appropriate definition of endpoints, (5) follow-up (>/< 5 years), (6) prospective study size assessment, and (7) patients included in follow-up/final analysis [19]. In cases of incomplete result presentation, studies were extra highlighted to assess risk of bias due to missing results [20]. Incongruences were resolved by consensus-reaching discussion of the subject by all authors.

### 2.6. Data Synthesis

To synthesize included studies, a narrative summary (outlining not only quantitative data, but also point/interval estimates if specified), combined with visualization of individual study characteristics, was presented. All data collected underwent no further modification. Given the significant methodological heterogeneity in terms of ki-67 assessment, we refrained from performing a meta-analysis of outcomes.

## 3. Results

**Figure 1:**
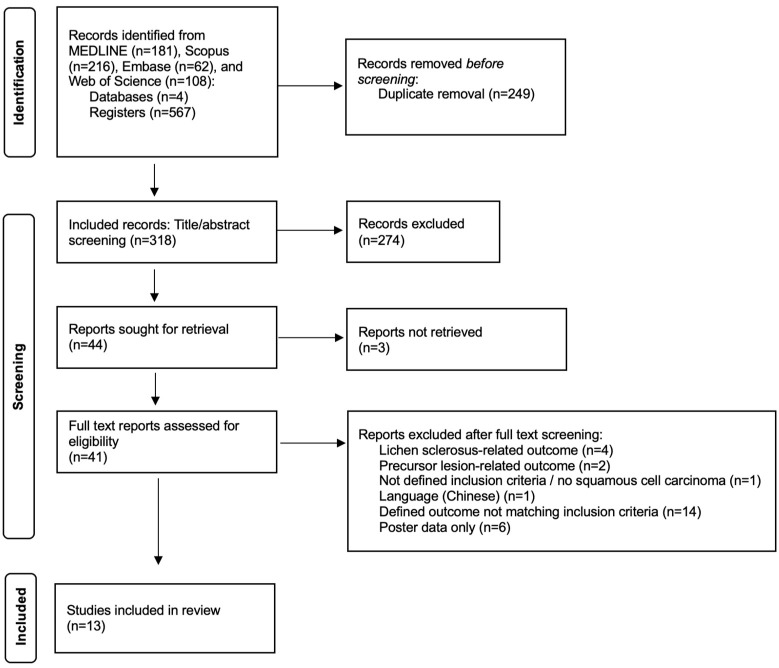
Flow diagram representing the study selection process.

**Figure 2:**
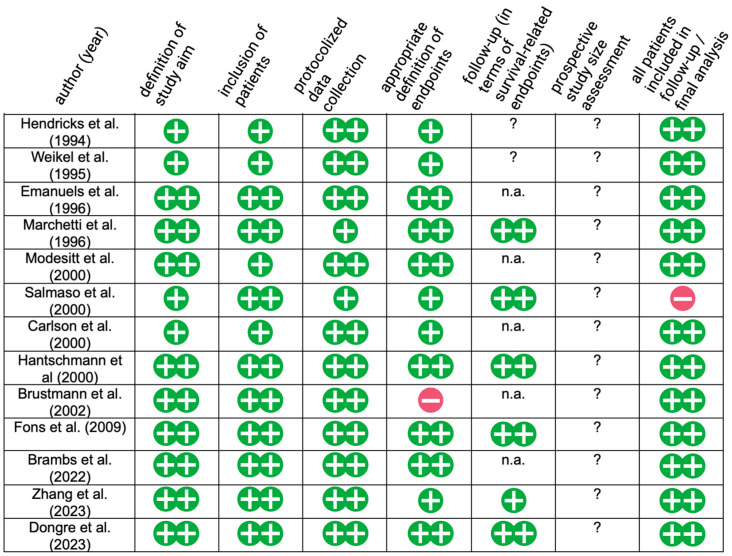
Risk of bias assessment listed for each study included ([49,50,51,52,53,54,55,56,57,58,59,60,61]) according to the MINORS tool: (++)—lowest risk of bias, (+)—low risk of bias, (?)—overall risk of bias unclear, (−)—potential risk of bias, n.a.—not applicable, in which case lymph node metastasis was defined as the only outcome.

In total, 567 database entries were retrieved, and 249 duplicates were removed. The abstracts of 318 records were screened using our a priori defined inclusion criteria, and 274 records were excluded, after which 44 publications were sought for retrieval and the full texts of 41 publications were assessed for eligibility; thereby, 28 reports were excluded ([21,22,23,24,25,26,27,28,29,30,31,32,33,34,35,36,37,38,39,40,41,42,43,44,45,46,47,48]) and 13 reports ([49,50,51,52,53,54,55,56,57,58,59,60,61]) were included in this study. See Figure 1 for a flow diagram presenting the study selection. Figure 2 presents the results of bias assessment. Regarding bias, 5 out of 13 studies were at low risk of bias, 6 were at middle risk, while 2 were at high risk. The technical details of the ki-67 staining protocols are depicted in Supplementary Table S3.

### 3.1. Interpretation of Positive Ki-67 Staining and Its Respective Cut-Off Values

To date, there is no common definition of ki-67 analysis and interpretation in vulvar cancer as a prognostic factor; in general, two approaches were determined. On the one hand, some researchers take a pattern-based approach, categorizing the distribution of ki-67-positive cells within the tumor mass into a distinct subgroup; on the other hand, there exists a subgroup division based on positive cell count in accordance with a defined amount of generally visible (tumor) cells.

**Figure 3:**
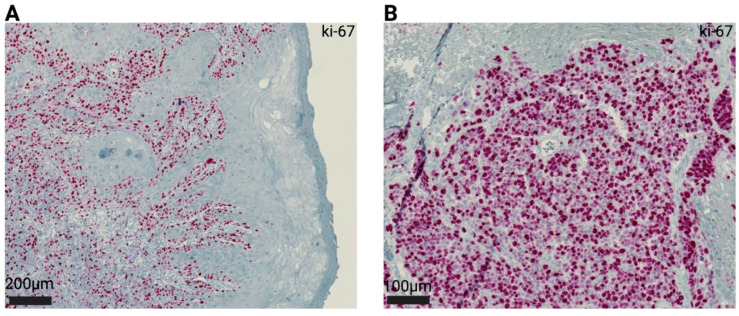
Representative presentation of the most frequently used patterns of ki-67 staining analysis within vulva carcinomas. (**A**): A “localized” distribution of ki-67-positive cells within basal tumor areas. (**B**): A “diffuse” pattern, with ki-67-positive cells scattered throughout the solid tumor mass.

Based on initial analysis of ki-67 pattern distributions in bladder cancer, as well as reports employing protease cathepsin-D and pro-cathepsin-L staining within a cohort of vulvar carcinomas, early studies defined two patterns of ki-67 staining on vulva carcinomas: a “localized” distribution of ki-67-positive nuclei restricted to basilar components of tumor clusters and a “diffuse” pattern, with ki-67-positive nuclei spreading universally all over the tumor mass [49,62,63]. Figure 3 shows both types of staining distributions in an exemplary manner. In 2000, Hantschmann et al. further subdivided the localized pattern. The already-defined localized pattern was named “basal type”; in case staining primarily occurs at the tumor invasion front, the pattern was defined as “infiltrative type” [56]. Brambs et al. (2022) [59] then again re-defined three patterns of ki-67 staining based on previous findings in penile squamous cell carcinoma and, in comparison to the physiological vulva epithelium: “Low-grade patterns” in the case of ki-67-positive nuclei were solely based on the outer cell layers of tumor clusters, “intermediate patterns” if ki-67-positive nuclei extended from the infiltrating zone to the tumor centrum, and “high-grade patterns” in the case of diffuse positive intratumoral staining [64].

In contrast, the ki-67 index is defined as the amount of positive stained cells per (total) number of tumor cells or, alternatively, per area [51]. Interpretation is widely based on a dichotomous approach, splitting up the study group in line with a priori defined cut-off values into a “ki-67-high group” and a “ki-67-low group”. In cases for which two subgroups were defined, the cut-off values <25%/>25% (Weikel et al.), as well as <50% / >50% (Zhang et al.), were established [50,60]. Another cut-off value was employed by Fons et al. (2009), who defined three groups according to the immunohistochemical cell count—“negative” in the case that less than 10% of nuclei stain positive, “weak positive” if up to 50% show positive nuclear staining, and “strong positive” if >50% of tumor nuclei are ki-67-positive [58].

Certain approaches employed both aforementioned methods: Marchetti et al. (1996) determined the number of ki-67-positive cells per 2000 tumor cells and also noted the specific staining pattern (diffuse/localized), subgrouping the latter into two distinct clusters (cut-off values: 0.2%–6% positivity / 7–9% positivity) [52]. Hantschmann et al. (2000) differentiated among three staining types (see above), but, moreover, divided their cohort into different proliferative indices (cut-off values: <10%, 11 to 50%, and >50%) by counting ki-67-positive cells per 200 tumor cells [56].

### 3.2. Association of Ki-67 with Lymph Node Metastasis

In total, ten studies examined the association of immunohistochemical ki-67 assessment with lymph node status; see Table 1 for a detailed realization. Hereby, only one group reported a statistically significant association, namely when analyzing the connotation between ki-67 staining pattern and lymph node metastasis (rho = 0.388, *p* = 0.0001; Spearman’s Rho and Cox Model) in relation to patient survival data [54], whereas all other studies confirmed no association. Since the interpretation of ki-67 staining varied widely (two patterns / three patterns / cell count), and negative study results were presented unbiased by the study population (variation: between 16 and 145 tumor cases), the observed effect (no correlation) seems to be independent of the analysis method per se.

### 3.3. Association of Ki-67 with Survival Data

Nine studies evaluated ki-67 as a prognostic marker in relation to overall survival (alternatively: disease-specific survival, with causes of death other than vulvar carcinoma excluded) and/or progression-free survival; refer to Table 2 for a comprehensive overview of study details. Whilst four studies found no statistically significant association of ki-67 with survival data, five studies determined differences in survival times based on ki-67 analysis. Although these mentioned studies all postulate a distinct connotation, with overall survival as their endpoint (only defined endpoint in terms of survival data of Marchetti et al. and Salmaso et al. [52,54]), an association with disease-free survival (progression-free survival) was not statistically significantly proven by Hantschmann et al. (*p* = 0.076), whereas it was, in contrast, statistically significant in the research by the groups of Dongre et al. (univariate analysis, *p* = 0.004) and Zhang et al. (*p* = 0.042, HR: 3.680) [56,60,61]. Regarding the mode of ki-67 analysis, only one of four studies employing the pattern-based approach determined no significant association between ki-67 analysis and survival data, whereas all three other studies did; percentage labeling of ki-67-positive nuclei or establishment of a proliferative index based on cell count (five studies in total) only proved successful in one study [61].

## 4. Discussion

Despite the tremendous progress in modern pathology—aiming for both precise, biologically determined tumor diagnostics, as well as a preferably unbiased and observer-independent approaches to tissue diagnostics—certain advantages result from continuously testing and reviewing standard and routine laboratory methods and markers such as ki-67. Due to their widespread use in clinical day-to-day practice, they hold potential in being a cheap, fast, and easily applicable tools, allowing for risk stratification in a global diagnostic setting, even though scientific discussions about the choice of the preferred antibody and threshold values in the evaluation of positive cells are still ongoing [65,66].

Within squamous cell carcinomas of the vulva, there is no established standard for the interpretation of proliferation-based immunohistochemical staining results. So far, each research team defined their own method and parameters of analysis; within our review, we could, in general, determine two semantically different approaches: a pattern-based approach and an analysis based on positive cell count. With regard to our a priori defined outcome parameters of interest, namely lymph node metastasis and survival data, we determined 13 studies meeting our inclusion criteria—as a limitation of our review, three studies ([67,68,69] potentially worthy of inclusion could not be retrieved even after contacting the authors and were thus not considered. We found convincing and broad evidence of a lacking statistically significant correlation—according to nine out of ten studies—between ki-67 staining and lymph node involvement in vulvar cancer. In contrast, the prognostic relevance of ki-67 immunohistochemistry regarding survival data was reported ambivalently in the literature—contradictory study results were published, although the majority of studies (five out of nine) postulated a prognostic impact of ki-67. Interestingly, four of the nine studies used a pattern-based method of analysis, three of which showed an association of ki-67 staining with overall survival, thus providing clear evidence of the preferrable method, aiming at sufficient evaluation of ki-67 immunohistochemistry in terms of prognosis in vulvar cancer. The remaining study, which also employed a pattern-based approach, but did not observe a statistically significant correlation, included the smallest number of participants (17 cases, in comparison to min. 73 patients in the other studies) and was therefore potentially underpowered [49,52]. From a histopathological point of view, a potential association between the degree of tumor cell differentiation and ki-67 distribution could be assumed. Indeed, within the examined study cohort, only the two oldest studies determined no significant correlation of tumor grade with a distinct ki-67 staining pattern (Hendricks et al. (*p* = 0.545) and Marchetti et al. [49,52]), whereas the remaining groups described an association of well-differentiated tumors with focal / basal staining patterns (Salmaso et al. (Spearman’s Rho = 0.344, *p* = 0.0001), and Hantschmann et al. (*p* = 0.024)) and consistently found a correlation of poor tumor differentiation with diffuse ki-67 staining pattern (Modesitt et al. (*p* = 0.013, CI 1.59 –7.60) [53,54,56]. In alignment, Brambs et al. employed the ki-67 staining pattern itself as a grading tool (low/intermediate/high grade pattern) in 2022 [59].

Within this area of research, our systematic review highlights several structural shortcomings, all of which naturally contribute to its limitations. Although the above-listed retrospective research projects all evaluated the prognostic impact of ki-67 staining in vulvar carcinoma, several studies included a small sample size (for example n = 16 or n = 17) [49,55]. That said, eligible studies show a distinct clinical (e.g., tumor stages included) and technical (e.g., different commercially available ki-67-clones provided by several manufacturers) heterogeneity, which should be carefully considered when interpreting a comparison between them. It is also striking that the studies included show a definite methodological heterogeneity, employing different strategies of statistical data analysis (see Table 1 and Table 2), which not only impacts comparability, but also yields a potential for bias. While some of these methodological differences may be explained by individual methods of data handling and analysis (e.g., interpretation of the type of variable, differences in data distribution within single studies), standardized methods of both data collection—the need for a defined cut-off arises from the diverse and wide range of cut-offs used in the studies reviewed here—and data analysis remain key within future study approaches. Such efforts would usually culminate in the establishment of international working groups that define an a priori standardized research design, aimed at producing reproducible and reliable results in histopathological research.

Finally, we were not able to perform subgroup analysis/interpretation because most of the included studies were performed prior to the current *2020 WHO Classification of Female Genital Tumors* and its classification of vulvar cancers based on their HPV-association/HPV-independence [70]. Future studies may even go beyond this and place the search for histological biomarkers in the context of the newly proposed molecular classification of vulvar cancer (HPV+/HPV-p53wildtype/HPV-p53mutant) [71]. Nevertheless, before results can be implemented into clinical routine, confirmation of the postulated evidence in ideally prospective, multicenter (to overcome interlaboratory variability), and regularly powered (to avoid statistical bias and allow further subgroup analysis) studies remains a fundamental necessity.

Breaking new methodological ground, the team of Zhang et al. was able to establish prognostic nomograms by using the parameters of age, HPV status, ki-67 index, PDL-1 analysis, and tumor-infiltrating t-cells. Thereby, the scoring of patients into two distinct groups could be established, proving a high concordance with survival data (overall survival *p* = 0.049, progression-free survival *p* = 0.0012) Additionally, future potential methodological improvements include the use of tissue micro arrays or automatic positive cell counts; in that regard, Fons et al. proved the valid use of either tissue micro arrays or full sections / full slide analysis in vulvar cancer ki-67 staining detection, and Choschzick et al. showed that deep convolutional neural network-based analysis was capable of detecting ki-67 indices (low <2%, medium 2–20%, high >20%), setting the base for less time-consuming and reliable data annotation [27,30]. That said, in the era of digital pathology, the implementation of novel tools, including artificial intelligence / machine learning may be not only less time consuming, but also more reproducible, contributing to further standardization of ki-67 interpretation by overcoming the high inter- and intra-observer variability reported, which probably arises due to a local heterogeneity in staining distribution, as well as varying color intensities within larger tumor sections [72]. Despite the aforementioned study by Choschzick et al., other research efforts in this field also aim to detect ki-67 using deep learning models in breast cancer or cervical intraepithelial lesions, or to develop a virtual ki-67-stained slide based solely on regular hematoxylin/eosin staining of squamous cell carcinomas [73,74,75]. Whether ki-67 could also play a contributing role as a distinct variable within a set of parameters used, e.g., in supervised machine learning approaches to calculate predictive probabilities in vulvar cancer, remains to be seen in future studies.

Beyond question, there are additional research interests making use of ki-67 examination in vulvar carcinoma. Thinking outside the box, Okon et al. aimed at addressing tumor heterogeneity; their unsupervised statistical approach using k-means clustering did determine different tumor sub-classes based on, among others, age and p53 expression, but not with statistically significant differences in ki-67-positively stained nuclei [26]. In another approach, ki-67 analysis was proposed to be associated with HPV status [21], and alternative immunohistochemical studies, despite ki-67 application, aim inter alia at p16 and p53, vascular endothelial growth factor, endothelial growth factor, estrogen receptor, HER2/neu, PD-L1 or epithelial–mesenchymal transition marker, and microsatellite marker detection / analysis [24,25,31,76,77,78]. Furthermore, ki-67 staining was evaluated as part of a dual staining approach in an immunocytochemistry study to distinguish between precursor lesions of the vulva and invasive vulvar neoplasia [48]. Two studies aimed at the comparison of ki-67 with alternative proliferative markers/activity; Brustmann et al. looked at nucleolar organizer regions and Topoisomerase II-alpha visualizations and did not determine a significant association of one of these proliferation markers with survival data; meanwhile, Emanuels et al. evaluated the ki-67 cell count, the proliferation marker Ag-NOR, and the mitotic index, but did not find an association of any proliferative markers with inguinofemoral lymph node metastases [51,57]. Whether prognostic impact can be derived from new proliferation markers—such as Phosphorylated Histone H3 (PHH3) staining, precisely solely mitotic cells and thereby holding potential to overcome invariances based on observer-dependent mitotic figure count—remains to be evaluated [79,80].

## 5. Conclusions

In summary, our systematic review depicts the postulated evidence of ki-67 as a potential prognostic marker in vulvar cancer. Although an association between ki-67 staining and lymph node metastasis could not be convincingly demonstrated, a positive association, in terms of survival data, was found in several studies, especially with the pattern-based analysis approach, despite study inconsistencies, such as different sizes of patient cohorts and varying ki-67 antibody suppliers. Considering the great availability of ki-67-stained vulvar cancers in global pathological archives, due to the method’s ubiquitous routine usage in daily diagnostics, even in low-income countries, additional retrospective analyses / evaluations of already-existing tissue samples seem reasonable in order to achieve more solid evidence of its prognostic impact. Moreover, prospective research efforts evaluating the prognostic connotation of ki-67 staining with survival data could be implemented in a potentially beneficial way with manageable cost and effort, utilizing this commonly employed routine diagnostic marker.

## Figures and Tables

**Table 1:** Depiction of studies examining the association of ki-67 and lymph node status. Significant associations are marked in bold.

Author (Year) and Number of Cases (n)	Method of Ki-67 Evaluation and Individual Cut-Off Value	Statistical Analysis	Results
Hendricks et al. (1994) [49]; n = 17	pattern: diffuse / localized positive nuclear area (PNA, quantification by automatic image analysis)	independent sample *t*-test	no association between lymph node status and ki-67 pattern (*p* = 963)
Emanuels et al. (1996) [51]; n = 145	Ki-67 index: positive nuclei per total number of nuclei (evaluation of 10 high-power fields)	Mann–Whitney U test	no relation between proliferation and lymph node metastases
Marchetti et al. (1996) [52]; n = 73	pattern: diffuse / localized (0.2–6% positivity / 7–9% positivity) pattern (hereby, ki-67 number of positive nuclei / 2000 neoplastic cells)	Fischer’s test	lymph node status is proposed to be correlated to ki-67 distribution (no statistical details provided)
Modesitt et al. (2000) [53]; n = 31	pattern: diffuse / localized	x^2^ and Fisher exact tests (required *p* was < 0.05)	no correlation between pattern and lymph node affect (not significant)
Salmaso et al. (2000) [54]; n = 129	pattern: diffuse / localized	Spearman’s Rho and Cox Model	**bivariate data analysis: significant association between ki-67 and OS with lymph node involvement (rho = 0.388, *p* = 0.0001)**
Carlson et al. (2000) [55]; n = 16	ki-67 labeling index: positive cells / 100 basal keratinocytes	ki-67 labeling index: positive cells / 100 basal keratinocytes x2 test: dichotomous variables t-test: continuous variables (equal variance) Mann–Whitney U test: continuous variables (unequal variance) correlation: linear regression analysis.	Elevated ki-67 was not associated with lymph node metastasis (*p* > 0.3)
Hantschmann et al. (2000) [56]; n = 74	pattern: diffuse type / infiltrating type / basal type Proliferative index: <10% / 11 to 50% / >50% (positive nuclei per 200 cells / 2)	X^2^-test (significance level of alpha = 0.05)	no association between ki-67 and lymph node metastases: basal type compared to diffuse and infiltrating types (*p* = 0.053)
Brustmann et al. (2002) [57]; n = 22	labeling indices: positive nuclei / 1000 epithelial cells	unpaired t-test and Pearson correlation (*p* < 0.05 was considered significant)	no association between ki-67 and development of lymph node metastases
Brambs et al. (2022) [59]; n = 32	pattern: low-grade / intermediate / high-grade	Mann–Whitney U test (2 groups) and Kruskal–Wallis test (>2 groups)	no significant association between ki-67 and lymph node metastases (*p* = 0.5)
Zhang et al. (2023) [60]; n = 69	ki-67 high vs. ki-67 low (cut-off value < or > 50% positive nuclei)	two-sided Fisher’s exact / Chi-square test as well as Cox regression (*p* < 0.05 was considered significant), Kaplan–Meier curves	no significant association between ki-67 and lymph node metastases (*p* = 0.672)

**Table 2:** Depiction of studies examining the association between ki-67 and survival data. Significant associations are marked in bold. Abbreviations: n.a.—not applicable (not defined).

Author (Year) and Number of Cases (n)	Method of Ki-67 Evaluation and Individual Cut-Off Value	Statistical Analysis	Results: Overall/Disease-Specific Survival	Results: Progression-Free/Disease-Free Survival
Hendricks et al. (1994) [49]; n = 17	pattern: diffuse / localized with their respective positive nuclear area (PNA, quantification by automatic image analysis)	Log-rank statistic to compare Kaplan–Meier survival curves	no association of ki-67 PNA (high vs. low *p* = 0.4193) with survival data	n.a.
Weikel et al. (1995) [50]; n = 147	MIB-1 <25% / >25%	univariate regression analysis	no statistically significant result for ki-67 staining (chi^2^ *p* = 0.11)	not distinctly reported
Marchetti et al. (1996) [52]; n = 73	pattern: diffuse / localized (0.2–6% positivity / 7–9% positivity) pattern (hereby, ki-67 number of positive nuclei / 2000 neoplastic cells)	Fischer’s test	**correlation of ki-67 patterns diffuse / local to survival after 3 years (*p* = 10^−8^) and 5 years (*p* = 5 × 10^−8^), but also difference in survival (localized low vs. localized high) at 3 years (*p* = 0.0025) and 5 years (0.00004)**	n.a.
Salmaso et al. (2000) [54]; n = 129	pattern: diffuse / localized	Spearman’s Rho and Cox Model (multivariate)	**After 5 years, 17% with a diffuse pattern were alive and 85% with a local pattern of staining were alive (Wilcoxon test, *p* = 0.001).** Multivariate analysis by Cox regression determines a **higher risk of death for ki-67- and p53-positive tumors vs. ki-67- and p53-negative tumors (RR: 4.29, 95% CI: 2.19–8.39, *p* = 0.00001)**	n.a.
Carlson et al. (2000) [55]; n = 16	ki-67 labeling index: positive cells / 100 basal keratinocytes	survival analysis: Cox proportional hazards model	no association between ki-67 and survival (all *p* > 0.3)	n.a.
Hantschmann et al. (2000) [56]; n = 74	pattern: diffuse type / infiltrating type / basal type Proliferative index: < 10% / 11 to 50% / > 50% (positive nuclei per 200 cells / 2)	Kaplan–Meier-analysis (log-rank-test and x^2^-test) and Cox regression for multivariate proportional analysis	Overall survival rates in the **diffuse and infiltrating types were statistically significantly reduced (*p* = 0.02),** with MIB-1 staining pattern as most valuable characteristic in multivariate analysis (odds ratio: 4.73, ***p* = 0.046**). The proliferation index analysis did not yield statistically significant results.	Diffuse and infiltrating types exhibited only a clinical tendency toward shorter disease-free survival (*p* = 0.076). The proliferation index analysis did not yield statistically significant results.
Fons et al. (2009) [58]; n = 80	negative (<10%), weak positive (10–50%), strong positive (>50%) staining	Kaplan–Meier product-limit method and Cox proportional hazard model as well as multivariate r/ univariate regression analysis	univariate analysis: Ki-67 >50% and disease-specific survival (*p* = 0.870, 95%CI = 0.30–2.73)	univariate analysis: Ki-67 >50% and disease-free survival (*p* = 0.297, 95%CI = 0.15–1.76)
Zhang et al. (2023) [60]; n = 69	ki-67 high vs. ki-67 low (cut-off value < or > 50% positive nuclei)	two-sided Fisher’s exact / Chi-square test as well as Cox regression (*p* < 0.05 was considered significant), Kaplan–Meier curves	univariate analysis: no association of ki-67 with overall survival (*p* = 0.448) **multivariate analysis:** **ki-67 high (*p* = 0.006, HR: 7.899) was significantly associated with decreased overall survival**	univariate analysis: no association of ki-67 with progression-free survival (*p* = 0.826) **multivariate analysis:** **ki-67 high (*p* = 0.042, HR: 3.680) was significantly associated with decreased progression-free survival**
Dongre et al. (2023) [61]; n = 123	ki-67 expression was dichotomized based on positive cells / 1000 tumor cells (percentage); cut-off was established by receiver operating curve (ROC)	Log-rank test and Kaplan–Meier curves, as well as Cox regression model	**univariate analysis:** **statistically significant association between ki-67 and disease specific survival (*p* = 0.041)** multivariate analysis: no impact of ki-67 on disease specific survival (*p* = 0.207)	**univariate analysis:** **significant association of ki-67 and progression-free survival (*p* = 0.004)** multivariate analysis: no association of ki-67 with progression-free survival (*p* = 0.158)

## Data Availability

The review protocol details, as well as extracted study data information, can be accessed on request; please refer to the corresponding author for individual agreements. The review was registered via OSF Registries [81].

## Supplementary Materials

The following supporting information can be downloaded at: https://www.mdpi.com/article/10.3390/jcm14062045/s1, Table S1: Search terms and fields for each database screened; Table S2: Overview of items employed in the initial “mixed-criteria” two-step approach assessing study quality; Table S3: Depiction of technical staining details within all included studies.

## References

[1] Gerdes J., Schwab U., Lemke H., Stein H. (1983). Production of a Mouse Monoclonal Antibody Reactive with a Human Nuclear Antigen Associated with Cell Proliferation. Int. J. Cancer.

[2] Scholzen T., Gerdes J. (2000). The Ki-67 Protein: From the Known and the Unknown. J. Cell Physiol..

[3] Kleer C.G., Giordano T.J., Braun T., Oberman H.A. (2001). Pathologic, Immunohistochemical, and Molecular Features of Benign and Malignant Phyllodes Tumors of the Breast. Mod. Pathol..

[4] Pirog E.C., Quint K.D., Yantiss R.K. (2010). P16/CDKN2A and Ki-67 Enhance the Detection of Anal Intraepithelial Neoplasia and Condyloma and Correlate with Human Papillomavirus Detection by Polymerase Chain Reaction. Am. J. Surg. Pathol..

[5] Vang R., Barner R., Wheeler D.T., Strauss B.L. (2004). Immunohistochemical Staining for Ki-67 and P53 Helps Distinguish Endometrial Arias-Stella Reaction from High-Grade Carcinoma, Including Clear Cell Carcinoma. Int. J. Gynecol. Pathol..

[6] Kolles H., Niedermayer I., Schmitt C., Henn W., Feld R., Steudel W.I., Zang K.D., Feiden W. (1995). Triple Approach for Diagnosis and Grading of Meningiomas: Histology, Morphometry of Ki-67/Feulgen Stainings, and Cytogenetics. Acta Neurochir..

[7] Tao Z., Xue R., Wei Z., Qin L., Bai R., Liu N., Wang J., Wang C. (2023). The Assessment of Ki-67 for Prognosis of Gastroenteropancreatic Neuroendocrine Neoplasm Patients: A Systematic Review and Meta-Analysis. Transl. Cancer Res..

[8] Nahed A.S., Shaimaa M.Y. (2016). Ki-67 as a Prognostic Marker According to Breast Cancer Molecular Subtype. Cancer Biol. Med..

[9] Gluz O., Nitz U., Christgen M., Braun M., Luedtke-Heckenkamp K., Darsow M., Forstbauer H., Potenberg J., Uleer C., Grischke E.-M. (2021). Prognostic Impact of Recurrence Score, Endocrine Response and Clinical-Pathological Factors in High-Risk Luminal Breast Cancer: Results from the WSG-ADAPT HR+/HER2- Chemotherapy Trial. J. Clin. Oncol..

[10] Nielsen T.O., Leung S.C.Y., Rimm D.L., Dodson A., Acs B., Badve S., Denkert C., Ellis M.J., Fineberg S., Flowers M. (2021). Assessment of Ki67 in Breast Cancer: Updated Recommendations from the International Ki67 in Breast Cancer Working Group. JNCI J. Natl. Cancer Inst..

[11] Ayhan A., Velipasaoglu M., Salman M.C., Guven S., Gultekin M., Bayraktar O. (2008). Prognostic Factors for Recurrence and Survival in Primary Vulvar Squamous Cell Cancer. Acta Obs. Gynecol. Scand..

[12] Fons G., Burger M.P., ten Kate F.J., van der Velden J. (2007). Identification of Potential Prognostic Markers for Vulvar Cancer Using Immunohistochemical Staining of Tissue Microarrays. Int. J. Gynecol. Pathol..

[13] Oonk M.H.M., Planchamp F., Baldwin P., Mahner S., Mirza M.R., Fischerová D., Creutzberg C.L., Guillot E., Garganese G., Lax S. (2023). European Society of Gynaecological Oncology Guidelines for the Management of Patients with Vulvar Cancer—Update 2023. Int. J. Gynecol. Cancer.

[14] Page M.J., McKenzie J.E., Bossuyt P.M., Boutron I., Hoffmann T.C., Mulrow C.D., Shamseer L., Tetzlaff J.M., Akl E.A., Brennan S.E. (2021). The PRISMA 2020 Statement: An Updated Guideline for Reporting Systematic Reviews. BMJ.

[15] Richardson W.S., Wilson M.C., Nishikawa J., Hayward R.S. (1995). The Well-Built Clinical Question: A Key to Evidence-Based Decisions. ACP J. Club.

[16] Eriksen M.B., Frandsen T.F. (2018). The Impact of Patient, Intervention, Comparison, Outcome (PICO) as a Search Strategy Tool on Literature Search Quality: A Systematic Review. J. Med. Libr. Assoc..

[17] Ouzzani M., Hammady H., Fedorowicz Z., Elmagarmid A. (2016). Rayyan—A Web and Mobile App for Systematic Reviews. Syst. Rev..

[18] Wortman P.M., Cooper H., Hedges L. (1994). Judging Research Quality. The Handbook of Research Synthesis.

[19] Slim K., Nini E., Forestier D., Kwiatkowski F., Panis Y., Chipponi J. (2003). Methodological Index for Non-randomized Studies (MINORS): Development and Validation of a New Instrument. ANZ J. Surg..

[20] Hayden J.A., Côté P., Bombardier C. (2006). Evaluation of the Quality of Prognosis Studies in Systematic Reviews. Ann. Intern. Med..

[21] Stewart C.J.R., Crook M.L. (2014). Fascin and Cyclin D1 Immunoreactivity in Non-Neoplastic Vulvar Squamous Epithelium, Vulvar Intraepithelial Neoplasia and Invasive Squamous Carcinoma: Correlation with Ki67 and P16 Protein Expression. J. Clin. Pathol..

[22] Cereceda K., Bravo N., Jorquera R., González-Stegmaier R., Villarroel-Espíndola F. (2022). Simultaneous and Spatially-Resolved Analysis of T-Lymphocytes, Macrophages and PD-L1 Immune Checkpoint in Rare Cancers. Cancers.

[23] Gualco M., Bonin S., Foglia G., Fulcheri E., Odicino F., Prefumo F., Stanta G., Ragni N. (2003). Morphologic and Biologic Studies on Ten Cases of Verrucous Carcinoma of the Vulva Supporting the Theory of a Discrete Clinico-Pathologic Entity. Int. J. Gynecol. Cancer.

[24] Lewy-Trenda I., Wierzchniewska-Ławska A., Papierz W. (2005). Expression of Vascular Endothelial Growth Factor (VEGF) in Vulvar Squamous Cancer and VIN. Pol. J. Pathol..

[25] Zannoni G.F., Prisco M.G., Vellone V.G., De Stefano I., Scambia G., Gallo D. (2011). Changes in the Expression of Oestrogen Receptors and E-Cadherin as Molecular Markers of Progression from Normal Epithelium to Invasive Cancer in Elderly Patients with Vulvar Squamous Cell Carcinoma. Histopathology.

[26] Okon K., Basta A., Stachura J. (1998). Morphometry Separates Squamous Cell Carcinoma of the Vulva into Two Distinct Groups. Pol. J. Pathol..

[27] Choschzick M., Alyahiaoui M., Ciritsis A., Rossi C., Gut A., Hejduk P., Boss A. (2021). Deep Learning for the Standardized Classification of Ki-67 in Vulva Carcinoma: A Feasibility Study. Heliyon.

[28] Mayer A., Schmidt M., Seeger A., Serras A.F., Vaupel P., Schmidberger H. (2014). GLUT-1 Expression Is Largely Unrelated to Both Hypoxia and the Warburg Phenotype in Squamous Cell Carcinomas of the Vulva. BMC Cancer.

[29] Zannoni G.F., Prisco M.G., Vellone V.G., de Stefano I., Vizzielli G., Tortorella L., Fagotti A., Scambia G., Gallo D. (2011). Cytoplasmic Expression of Oestrogen Receptor Beta (ERβ) as a Prognostic Factor in Vulvar Squamous Cell Carcinoma in Elderly Women. Histopathology.

[30] Fons G., Van Der Velden J., Burger M., Ten Kate F. (2009). Validation of Tissue Microarray Technology in Vulvar Cancer. Int. J. Gynecol. Pathol..

[31] Brustmann H. (2007). Epidermal Growth Factor Receptor Is Involved in the Development of an Invasive Phenotype in Vulvar Squamous Lesions, but Is Not Related to MIB-1 Immunoreactivity. Int. J. Gynecol. Pathol..

[32] Nola M., Blažanovíc A., Dotlić S., Morović A., Tomičić I., Jukić S. (2005). Invasive Squamous Cell Carcinoma of Vulva: Prognostic Significance of Clinicopathologic Parameters. Croat. Med. J..

[33] Koyamatsu Y., Yokoyama M., Nakao Y., Fukuda K., Saito T., Matsukuma K., Iwasaka T. (2003). A Comparative Analysis of Human Papillomavirus Types 16 and 18 and Expression of P53 Gene and Ki-67 in Cervical, Vaginal, and Vulvar Carcinomas. Gynecol. Oncol..

[34] Biatta C.M., Paudice M., Scaglione G., Baldelli I., Fulcheri E., Ferrero S., Cozzani E., Parodi A., Vellone V.G. (2020). Molecular Markers Expression in the Transition from Normal Epithelium to Invasive Cancer in Vulvar Squamous Cell Carcinoma. J. Dermatol. Nurses’ Assoc..

[35] Sznurkowski J., Zawrocki A., Sznurkowska K., Biernat W. (2017). Ki67 and CD44 indices depends on P16 status of vulvar cancer tissue. High index of CD44 positive cancer cells predicts worse outcome of patients. Int. J. Gynecol. Cancer.

[36] Stefansson K., Oda H., Öfverman C., Lundin E., Hedman H., Lindquist D. (2015). LRIG Protein Expression and HPV Prevalence in Vulvar Cancer Patients in Northern Sweden. Int. J. Gynecol. Cancer.

[37] Novikova E., Chulkova O., Filonenko E. (2012). P53 and KI-67 Expression in a Premalignant Lesions and Cancer of Vulva. Int. J. Gynecol. Cancer.

[38] Novikova E.G., Chulkova O.V., Filonenko E.V., Murshudova S.K. (2011). P53 and KI-67 Expression in Vulvar Carcinoma, Vulvar Intraepithelial Neoplasia, Squamous Cell Hiperplasia and Lichen Sclerosus. Int. J. Gynecol. Cancer.

[39] Hampl M., Panyatopoulos D., Roos J., Petry H.U., Dürst M., von Knebel Döberitz M., Reuschenbach M. (2010). The Small Vulvar Cancer of the Anterior Fourchette: A Specific Tumor Entity?. Arch. Gynecol. Obs..

[40] Carlson J.A., Ambros R., Malfetano J., Ross J., Grabowski R., Lamb P., Figge H., Mihm M.C. (1998). Vulvar Lichen Sclerosus and Squamous Cell Carcinoma: A Cohort, Case Control, and Investigational Study with Historical Perspective; Implications for Chronic Inflammation and Sclerosis in the Development of Neoplasia. Hum. Pathol..

[41] Drew P.A., Malik S.N., Wilkinson E.J. (1998). Laminin, Bcl-2 Protein and Ki-67 Expression in Vulvar Carcinoma. Mod. Pathol..

[42] Scurry J., Beshay V., Cohen C., Allen D. (1998). Ki67 Expression in Lichen Sclerosus of Vulva in Patients with and without Associated Squamous Cell Carcinoma. Histopathology.

[43] Ben-Hur H., Ashkenazi M., Huszar M., Gurevich P., Zusman I. (2001). Lymphoid Elements and Apoptosis-Related Proteins (Fas, Fas Ligand, P53 and Bcl-2) in Lichen Sclerosus and Carcinoma of the Vulva. Eur. J. Gynaecol. Oncol..

[44] Al-Ghamdi A., Freedman D., Miller D., Poh C., Rosin M., Zhang L., Gilks C.B. (2002). Vulvar Squamous Cell Carcinoma in Young Women: A Clinicopathologic Study of 21 Cases. Gynecol. Oncol..

[45] Carlson J.A., Amin S., Malfetano J., Tien A.T., Selkin B., Hou J., Goncharuk V., Wilson V.L., Rohwedder A., Ambros R. (2001). Concordant P53 and Mdm-2 Protein Expression in Vulvar Squamous Cell Carcinoma and Adjacent Lichen Sclerosus. Appl. Immunohistochem. Mol. Morphol..

[46] Tessier-Cloutier B., Asleh-Aburaya K., Shah V., McCluggage W.G., Tinker A., Gilks C.B. (2017). Molecular Subtyping of Mammary-like Adenocarcinoma of the Vulva Shows Molecular Similarity to Breast Carcinomas. Histopathology.

[47] Wei L.-B., Pu D.-M., Yin L. (2008). Relationship between DNA ploidy and cell proliferation of keratinizing vulvar squamous cell carcinomas and non-neoplastic epithelial disorders. Chin. J. Cancer Prev. Treat..

[48] Takacs F.Z., Radosa J.C., Bochen F., Juhasz-Böss I., Solomayer E.-F., Bohle R.M., Breitbach G.-P., Schick B., Linxweiler M. (2019). Sec62/Ki67 and P16/Ki67 Dual-Staining Immunocytochemistry in Vulvar Cytology for the Identification of Vulvar Intraepithelial Neoplasia and Vulvar Cancer: A Pilot Study. Arch. Gynecol. Obstet..

[49] Hendricks J.B., Wilkinson E.J., Kubilis P., Drew P., Blaydes S.M., Munakata S. (1994). Ki-67 Expression in Vulvar Carcinoma. Int. J. Gynecol. Pathol..

[50] Weikel W., Beck T., Moll R., Brutnm C., Knapstein P.G. (1995). Prognostic evaluation of vulvar carcinomas: Histological, immunohistochemical, and flow-cytometric investigations. Gynakol. Geburtshilfliche Rundsch..

[51] Emanuels A.G., Burger M.P.M., Hollema H., Koudstaal J. (1996). Quantitation of Proliferation-Associated Markers Ag-NOR and Ki-67 Does Not Contribute to the Prediction of Lymph Node Metastases in Squamous Cell Carcinoma of the Vulva. Hum. Pathol..

[52] Marchetti M., Salmaso R., Polonio S., Perin D., Salviato T., Onnis A. (1996). Ki-67 Expression in Vulvar Carcinoma. Preliminary Results. Eur. J. Gynaecol. Oncol..

[53] Modesitt S.C., Groben P.A., Walton L.A., Fowler W.C., Van Le L. (2000). Expression of Ki-67 in Vulvar Carcinoma and Vulvar Intraepithelial Neoplasia III: Correlation with Clinical Prognostic Factors. Gynecol. Oncol..

[54] Salmaso R., Zen T., Zannol M., Perin D., Marchiori S., Marchetti M. (2000). Prognostic Value of Protein P53 and Ki-67 in Invasive Vulvar Squamous Cell Carcinoma. Eur. J. Gynaecol. Oncol..

[55] Carlson J.A., Healy K., Tran T.A., Malfetano J., Wilson V.L., Rohwedder A., Ross J.S. (2000). Chromosome 17 Aneusomy Detected by Fluorescence in Situ Hybridization in Vulvar Squamous Cell Carcinomas and Synchronous Vulvar Skin. Am. J. Pathol..

[56] Hantschmann P., Lampe B., Beysiegel S., Kurzl R. (2000). Tumor Proliferation in Squamous Cell Carcinoma of the Vulva. Int. J. Gynecol. Pathol..

[57] Brustmann H., Naudé S. (2002). Expression of Topoisomerase IIα, Ki-67, Proliferating Cell Nuclear Antigen, P53, and Argyrophilic Nucleolar Organizer Regions in Vulvar Squamous Lesions. Gynecol. Oncol..

[58] Fons G., Burger M.P.M., Ten Kate F.J.W., Van Der Velden J. (2009). Assessment of Promising Protein Markers for Vulva Cancer. Int. J. Gynecol. Cancer.

[59] Brambs C.E., Horn L.-C., Mende M., Höckel M., Eckey C., Hiller G.G.R., Höhn A.K. (2022). Epithelial–Mesenchymal Transition (EMT) in Vulvar Cancer with and without Inguinal Lymph Node Involvement. J. Cancer Res. Clin. Oncol..

[60] Zhang T., Zhu Y., Luo J., Li J., Niu S., Chen H., Zhou F. (2023). An Integrated Model for Prognosis in Vulvar Squamous Cell Carcinoma. BMC Cancer.

[61] Dongre H.N., Elnour R., Tornaas S., Fromreide S., Thomsen L.C.V., Kolseth I.B.M., Nginamau E.S., Johannessen A.C., Vintermyr O.K., Costea D.E. (2023). TP53 Mutation and Human Papilloma Virus Status as Independent Prognostic Factors in a Norwegian Cohort of Vulva Squamous Cell Carcinoma. Acta Obstet. Gynecol. Scand..

[62] Fontana D., Bellina M., Gubetta L., Fasolis G., Rolle L., Scoffone C., Porpiglia F., Colombo M., Tarabuzzi R., Leonardo E. (1992). Monoclonal Antibody Ki-67 in the Study of the Proliferative Activity of Bladder Carcinoma. J. Urol..

[63] Hantschmann P., Beysiegel S., Kürzl R. (1998). Squamous Cell Carcinoma of the Vulva. Expression of Tumor Proteases Cathepsin-D and pro-Cathepsin-L. J. Reprod. Med..

[64] Stankiewicz E., Ng M., Cuzick J., Mesher D., Watkin N., Lam W., Corbishley C., Berney D.M. (2012). The Prognostic Value of Ki-67 Expression in Penile Squamous Cell Carcinoma. J. Clin. Pathol..

[65] Lindboe C.F., Torp S.H. (2002). Comparison of Ki-67 Equivalent Antibodies. J. Clin. Pathol..

[66] Geread R.S., Morreale P., Dony R.D., Brouwer E., Wood G.A., Androutsos D., Khademi A. (2019). IHC Color Histograms for Unsupervised Ki67 Proliferation Index Calculation. Front. Bioeng. Biotechnol..

[67] Emanuels A., Hollema H., Suurmeyer A., Koudstaal J. (1994). A Modified Method for Antigen Retrieval MIB-1 Staining of Vulvar Carcinoma. Eur. J. Morphol..

[68] Abrao F.S., Baracat E.C., Marques A.F., Abrao M.S., Torloni H., Coelho F.R.G., Alves A.C., De Lima G.R. (1990). Carcinoma of the Vulva: Clinicopathologic Factors Involved in Inguinal and Pelvic Lymph Node Metastasis. Proc. J. Reprod. Med. Obstet. Gynecol..

[69] Hoffmann G., Casper F., Weikel W., Kümmerle T., Pollow B., Schaffrath M., Hofmann M., Pollow K. (1999). Prognostic value of p53, UPA, PAI-1 and Ki-67 in vulvar carcinoma. Zentralbl Gynakol..

[70] Lokuhetty D., White V.A., Watanabe R. (2020). Female Genital Tumours. WHO Classification of Tumours.

[71] Kortekaas K.E., Bastiaannet E., van Doorn H.C., de Vos van Steenwijk P.J., Ewing-Graham P.C., Creutzberg C.L., Akdeniz K., Nooij L.S., van der Burg S.H., Bosse T. (2020). Vulvar Cancer Subclassification by HPV and P53 Status Results in Three Clinically Distinct Subtypes. Gynecol. Oncol..

[72] Shui R., Yu B., Bi R., Yang F., Yang W. (2015). An Interobserver Reproducibility Analysis of Ki67 Visual Assessment in Breast Cancer. PLoS ONE.

[73] Phan T.-C., Huu H.N. (2024). Enhancing Breast Cancer Detection with Advanced Deep Learning Techniques for Ki-67 Nuclear Protein Analysis. SN Comput. Sci..

[74] Martino F., Ilardi G., Varricchio S., Russo D., Di Crescenzo R.M., Staibano S., Merolla F. (2024). A Deep Learning Model to Predict Ki-67 Positivity in Oral Squamous Cell Carcinoma. J. Pathol. Inform..

[75] Sheikhzadeh F., Ward R.K., van Niekerk D., Guillaud M. (2018). Automatic Labeling of Molecular Biomarkers of Immunohistochemistry Images Using Fully Convolutional Networks. PLoS ONE.

[76] Garganese G., Inzani F., Fragomeni S.M., Mantovani G., Della Corte L., Piermattei A., Santoro A., Angelico G., Giacò L., Corrado G. (2021). The Vulvar Immunohistochemical Panel (VIP) Project: Molecular Profiles of Vulvar Squamous Cell Carcinoma. Cancers.

[77] Woelber L., Prieske K., Eulenburg C., Oliveira-Ferrer L., de Gregorio N., Klapdor R., Kalder M., Braicu I., Fuerst S., Klar M. (2021). P53 and P16 Expression Profiles in Vulvar Cancer: A Translational Analysis by the Arbeitsgemeinschaft Gynäkologische Onkologie Chemo and Radiotherapy in Epithelial Vulvar Cancer Study Group. Am. J. Obs. Gynecol..

[78] Rodrigues I.S., Lavorato-Rocha A.M., de M Maia B., Stiepcich M.M.A., de Carvalho F.M., Baiocchi G., Soares F.A., Rocha R.M. (2013). Epithelial-Mesenchymal Transition-like Events in Vulvar Cancer and Its Relation with HPV. Br. J. Cancer.

[79] Kim J.-Y., Jeong H.S., Chung T., Kim M., Lee J.H., Jung W.H., Koo J.S. (2017). The Value of Phosphohistone H3 as a Proliferation Marker for Evaluating Invasive Breast Cancers: A Comparative Study with Ki67. Oncotarget.

[80] Elmaci İ., Altinoz M.A., Sari R., Bolukbasi F.H. (2018). Phosphorylated Histone H3 (PHH3) as a Novel Cell Proliferation Marker and Prognosticator for Meningeal Tumors: A Short Review. Appl. Immunohistochem. Mol. Morphol..

[81] Klamminger G.G., Nigdelis M. (2024). Ki-67 as a Prognostic Marker in Squamous Cell Carcinoma of the Vulva. https://osf.io/9n5es/.

