# Supplementary material for: Ki-67 as a Prognostic Marker in Squamous Cell Carcinomas of the Vulva: A Systematic Review
Source: J Clin Med. 2025 Mar 17;14(6):2045. doi: 10.3390/jcm14062045 (PMC11942767; doi:10.3390/jcm14062045)
Supplement: Supplementary file 1 [file jcm-14-02045-s001.zip › jcm-3505010-supplementary.pdf]

Supplementary Material

The PICO framework for this study is defined as:

Population: HPV associated/independent squamous cell carcinomas of the vulva

Intervention: immunohistochemical ki-67 stainings

Comparison: recognition of various methods of Ki-67 reporting/interpretation, group comparison based on different ways ki-67 analyses (pattern based vs. cell-count based)

Outcome: occurrence of lymph node metastasis, association of ki-67 staining with overall/progression free survival

**Database**	**Search terms**
MEDLINE	((((((((Vulvar Neoplasms) OR (Vulvar Neoplasms[MeSH Terms])) OR (vulvar cancer)) OR (vulvar carcinoma)) OR (vulvar tumor)) OR (vulvar tumour)) OR (vulvar malignancy)) OR (vulvar malignant))) AND ((((((((Ki-67)) OR (Ki67)) OR (MIB-1)) OR (MIB1)) OR (proliferative index)) OR (proliferative activity)) OR (mitotic index')) OR (mitotic count))<br>[ALL FIELDS]
Scopus	(vulvar Neoplasms or vulvar cancer or vulvar carcinoma or vulvar tumor or vulvar tumour or vulvar malignancy or vulvar malignant) and (Ki-67 or MIB-1 or MIB1 or proliferative index or proliferative activity or mitotic index or mitotic count)<br>Search within: Article title, Abstract, Keywords
Embase	(((vulvar neoplasm or vulvar cancer or vulvar carcinoma or vulvar tumor or vulvar tumour or vulvar malignancy or vulvar malignant) and (Ki-67 or Ki67 or MIB1 or MIB-

	1 or proliferative index or proliferative activity or mitotic index or mitotic count))).af
Web of Science	ALL=((vulvar neoplasm OR vulvar cancer OR vulvar carcinoma OR vulvar tumor OR vulvar tumour OR vulvar malignancy OR vulvar malignant) AND (Ki-67 OR Ki67 OR MIB1 OR MIB-1 OR proliferative index OR proliferative activity OR mitotic index OR mitotic count))

Supplemental Table S1. Search terms and fields for each database screened.

**Quality item(s)**	**Characteristics (yes/partly/no/unsure)**
Characteristics of the study population are adequately described (number of tumors, histology, histo-pathological features, period and area of recruitment).	**Study participation**: the study sample (patients / tumors included) is characteristic for the population of interest.
Supplier of the ki-67 antibody, mode and thresholds of ki67 detection are defined.	**Prognostic factor measurement**: The variable of interest and its detection / interpretation mode is clearly described.
Outcome is clearly defined, data of follow up is provided, study setting is applicable to all participants.	**Outcome measurement**: The outcome is measured adequately.
Data is presented and visualized without selective results reporting, way and methods of data analysis fit the study design.	**Analysis**: Alignment of statistical method and study design.

Supplemental Table S2. Overview of items employed in the initial “mixed-criteria” two-step approach assessing study quality.

**author** (year)	**antibody information**	**tissue type / fixation method (e.g., formalin fixed paraffin embedded; FFPE)**	**direct/in-direct method of immunohistochemistry**	**type of antibody detection; chromogen information**
Hendricks et al. (1994)	MIB-1 antibody (AMAC, Inc., Westbrook, ME, US,); dilution 1:20	FFPE tissue (4 µm)	indirect	biotin-streptavidin detection system; DAB served as chromagen (Bio-Genex, San Ramon, CA)
Weikel et al. (1995)	MIB-1, (Dianova, Hamburg, Germany,); dilution 1:50	n.a.	n.a.	n.a.
Emanuels et al. (1996)	MIB-1 antibody (Dianova, Hamburg, Germany)	FFPE tissue (4 µm)	indirect	Biogenex kit (San Ramon, CA): antimouse biotin and alkaline phosphatase conjugated streptavidin
Marchetti et al. (1996)	ki-67 - n.a.	FFPE tissue	n.a.	n.a.
Modesitt et al. (2000)	MIB-1 (mouse monoclonal; Immunotech, Westbrook, ME, US)	FFPE tissue (4 µm)	indirect	streptavidin–biotin detection system; DAB served as chromagen (DAKO, Carpinteria, CA)
Salmaso et al. (2000)	ki-67 - n.a.	FFPE tissue	n.a.	n.a.
Carlson et al. (2000)	Mib-1 (Ventana Medical Systems)	10% standard buffered formalin followed by routine processing	n.a.	Ventana ES automated diaminobenzidine immunohistochemical system (Ventana Medical Systems, Tucson, AZ)
Hantschmann et al (2000)	MIB-1 (Dianova, Hamburg, Germany); dilution 1:100	FFPE tissue	indirect	detection method: avidin-biotin-complex and peroxidase-DAB
Brustmann et al. (2002)	affinity-isolated prediluted ready-to-use ki-67 antibody (rabbit)	FFPE tissue (3 µm); formalin fixation did not exceed 24 hours.	indirect	LSAB2 (labeled streptavidin–biotin) kit, alkaline phosphatase (Dako Corp., Carpinteria, CA)
Fons et al. (2009)	MIB-1 (Dako Omnis); dilution 1:200	FFPE tissue	indirect	avidin- biotin method
Brambs et al. (2022)	MIB-1 (Dako Omnis); dilution 1:100	n.a.; most likely employment of FFPE tissue	n.a.	detection system: DAB
Zhang et al. (2023)	Ki-67 (GM001, GeneTech)	FFPE tissue (4 µm)	indirect	EnVision™ FLEX Mouse LINKER anti-mouse linker antibody + EnVision™ FLEX HRP visualization reagent + DAB chromogen (+ enhancer)
Dongre et al. (2023)	anti-Ki67 (clone MIB-1; Dako-Agilent); dilution 1:100	FFPE tissue	indirect	BrightVision Ultimate plus goat anti-mouse/rabbit HRP-DAB kit (Medac Diagnostics)

Supplemental Table S3. Depiction of technical staining details within all included studies. DAB=diaminobenzidine; n.a.=not available: information not assessable within the original manuscript.
